# Direct Tracking of Charge Carrier Drift and Extraction from Perovskite Solar Cells by Means of Transient Electroabsorption Spectroscopy

**DOI:** 10.1021/acsaelm.2c01346

**Published:** 2023-01-11

**Authors:** Vidmantas Jašinskas, Marius Franckevičius, Andrius Gelžinis, Jevgenij Chmeliov, Vidmantas Gulbinas

**Affiliations:** † Department of Molecular Compound Physics, Center for Physical Sciences and Technology, Saulėtekio av. 3, VilniusLT-10257, Lithuania; ‡ Institute of Chemical Physics, Faculty of Physics, Vilnius University, Saulėtekio av. 9, VilniusLT-10222, Lithuania

**Keywords:** perovskite solar cell, carrier, mobility, electroabsorption, ultrafast spectroscopy

## Abstract

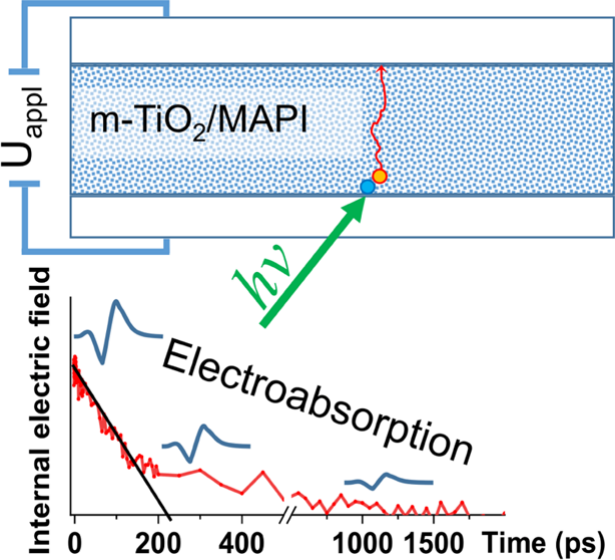

The best perovskite solar cells currently demonstrate more than 25% efficiencies, yet many fundamental processes that determine the operation of these devices are still not fully understood. In particular, even though the device performance strongly depends on charge carrier transport across the perovskite layer to selective electrodes, information about this process is still very controversial. Here, we investigate charge carrier motion and extraction from an archetypical CH_3_NH_3_PbI_3_ (MAPI) perovskite solar cell. We use the ultrafast electric-field-modulated transient absorption technique, which allows us to evaluate the electric field dynamics from the time-resolved electroabsorption spectra and to visualize the motion of charge carriers with subpicosecond time resolution. We demonstrate that photogenerated holes drift across the mesoporous TiO_2_/perovskite layer during hundreds of picoseconds. On the other hand, their extraction into the spiro-OMeTAD hole transporting layer lasts for more than 1 nanosecond, suggesting that the hole extraction is limited by the perovskite/spiro-OMeTAD interface rather than by the hole transport through the perovskite layer. Additionally, we use the ultrafast time-resolved fluorescence technique that reveals fluorescence decay during tens of picoseconds, which we attribute to the spatial separation of electrons and holes.

## Introduction

Charge carrier motion is one of the most important processes determining the performance of all electronic devices, including perovskite solar cells (PSCs). Typically, the transport properties of charge carriers in semiconductors are characterized by carrier mobility and diffusivity. These two parameters are interrelated by the classical Einstein relation, which, however, has its validity limitations, especially for disordered and inhomogeneous materials.^1−3^ In the case of perovskites, carrier mobility and diffusivity still remain poorly characterized and understood. The reported mobility values vary by many orders of magnitude depending on the material preparation, device architecture, and measurement techniques.^4^ Moreover, the sample configurations required for mobility studies are often very different from those used in real devices, which makes the applicability of obtained mobility values questionable. For example, contactless THz or microwave techniques probe carrier motion in thin films formed on quartz substrates. This probing is done on very short distances and typically yields high mobility values reaching tens of cm^2^·V^–1^·s^–1^.^5−7^ On the other hand, much lower mobility values of about 1 cm^2^·V^–1^·s^–1^ were obtained in thin MAPI films using the photoluminescence quenching technique that probes carrier motion perpendicular to the film surface.^8^ Other commonly used techniques for probing carrier motion in sandwich-type device configurations, such as time-of-flight (TOF) or charge extraction by linear increasing voltage (CELIV), usually yield similar or even orders of magnitude lower mobility values.^9−11^ These large differences were explained by the morphology of perovskite films, where grain boundaries, lattice defects, and carrier traps cause significantly different short-distance and long-distance carrier mobilities and are thus strongly dependent on the way carrier mobilities are measured.^12^ It has also been suggested that carrier mobilities are time-dependent because of the confinement of charge carriers within the fractal-like spatial network during nanoseconds.^9^

Consequently, carrier motion in real perovskite solar cells still remains far from clear and the lack of appropriate techniques to study charge carrier mobility is one of the major problems. Moreover, the evaluation of the actual electric field strength in the perovskite layer of solar cells is also not a trivial task. Therefore, the charge carrier transport in real operating perovskite solar cells still remains a controversial, heavily disputed question.^13,14^ Carrier motion is expected to be particularly complex in the case of the archetypical perovskite solar cell architecture, where majority of the perovskite is embedded into the mesoporous TiO_2_ (m-TiO_2_) layer.

The carrier transport is closely related to another important process—carrier extraction from the perovskite layer to transport layers. Fast carrier extraction, which reduces charge carrier density in the perovskite layer and wins competition with the recombination, is believed to be one of the major factors determining power conversion efficiency and also affecting device degradation. It is still not clear to what extent charge carrier extraction is determined by the carrier transport and the properties of the interface between the perovskite and the transporting layer, where both the trap states and the barriers may hinder the carrier extraction.

In this work, we study the carrier motion and extraction dynamics in an archetypical MAPI perovskite solar cell by combining ultrafast transient absorption and fluorescence techniques, additionally modulating their signals by an applied external voltage. We evaluate the carrier motion dynamics from the evolution of the electroabsorption spectra of photoexcited solar cells, while conventional transient absorption provides information on carrier extraction. We demonstrate that carrier motion across the m-TiO_2_/perovskite layer takes place during hundreds of picoseconds under the applied voltage of several volts, while carrier extraction occurs on a nanosecond time scale.

## Materials and Methods

### Solar Cell Preparation

In this study, we have investigated an archetypical MAPI perovskite solar cell fabricated by a two-step sequential interdiffusion technique.^15^ A compact TiO_2_ layer of about 30–40 nm thickness was deposited on the transparent FTO-coated glass substrates followed by spray pyrolysis of ethanol (4.5 mL) containing titanium diisopropoxide bis(acetylacetonate) (0.3 mL, 75% in 2-propanol, Sigma-Aldrich) and acetylacetone (0.2 mL, ≥99%, Sigma-Aldrich) at 450 °C in air. On top of this layer, mesoporous titanium dioxide with a thickness of about 300 nm was formed by spin-coating TiO_2_ nanoparticles (30 nm sized, 30NRT, Dyesol) diluted in ethanol (≥99.8%, Sigma-Aldrich) (1:3.5 w/w) at 4800 r.p.m. for 20 s. The formed layer was heated up to 500 °C for 1 h in an ambient atmosphere. Afterward, the stock solution of lead iodide (PbI_2_) (1.2 M, 99.99%, TCI Chemicals) in *N,N*-anhydrous dimethylformamide (99.8%, Acros) was spin-coated on top of the mesoporous TiO_2_ layer at 6500 r.p.m. for 20 s and dried for 15 min at 100 °C. The deposition of lead iodide was repeated two times. Then, CH_3_NH_3_I in isopropanol solution (0.05 M) was sprayed on top of the deposited PbI_2_ layer and left for 20 s before spin coating at 4000 r.p.m. for 20 s and dried at 80 °C for 15 min. Subsequently, about 200 nm layer of spiro-MeOTAD was deposited as a hole transporting material on the formed perovskite films by spin coating at 3000 rpm for 20 s. The devices were terminated by thermally evaporating a 100 nm thick gold layer.

**Figure 1:**
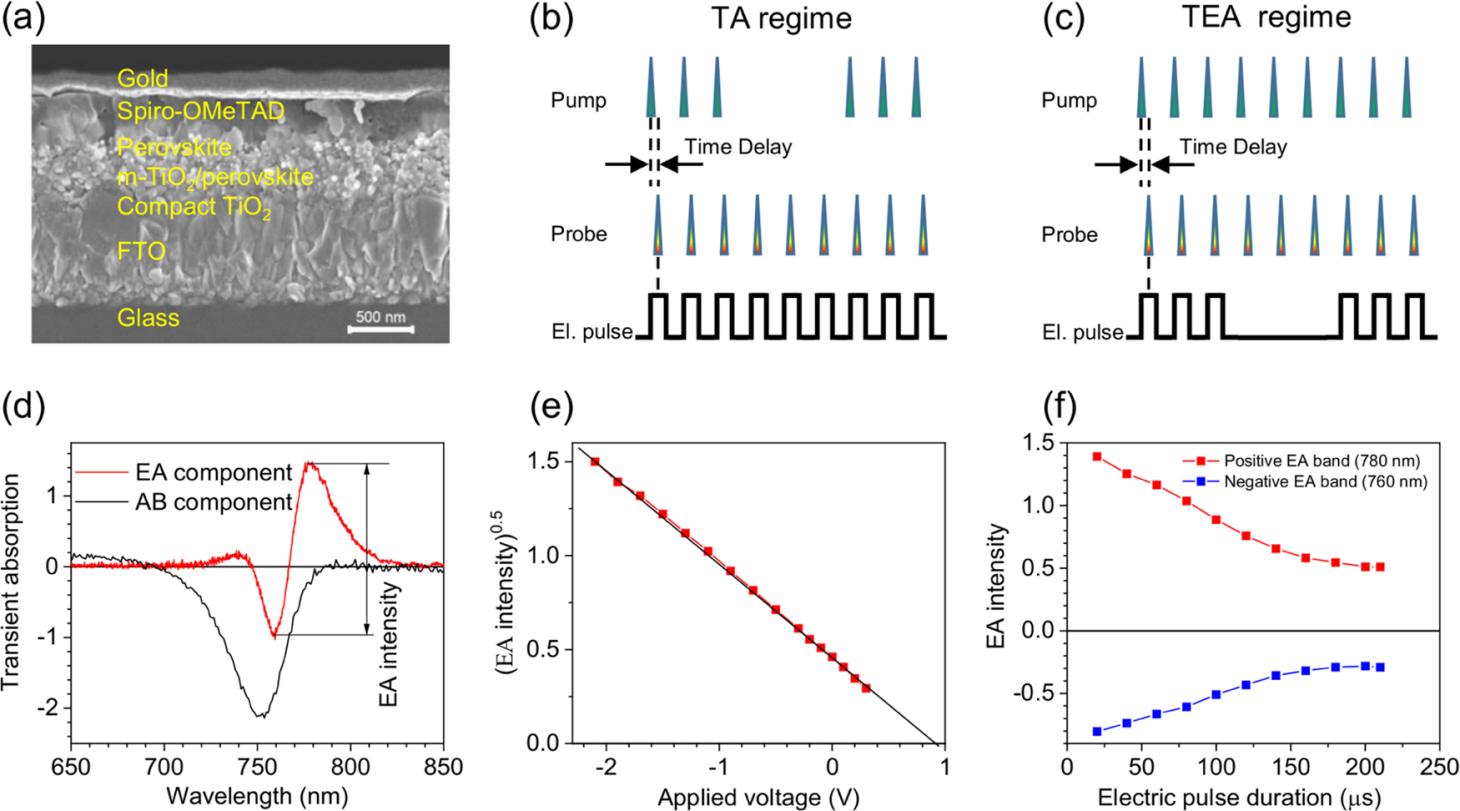
(a) SEM image of the investigated MAPI solar cell; (b) pulse timing scheme in the conventional transient absorption (TA) regime and (c) in transient electroabsorption (TEA) regime; (d) conventional transient absorption spectrum (AB component) measured under a forward 1 V bias, compensating the built-in electric field (black curve) and electroabsorption spectrum (EA component) measured under a −1 V bias without optical excitation (red curve); the vertical bar shows how the EA intensity was evaluated; (e) square root of the EA intensity as a function of the applied voltage; and (f) amplitudes of the positive and negative EA bands as functions of the duration of the applied voltage pulses.

The cross-sectional SEM image of the investigated solar cell is shown in Figure 1a. In this architecture, the active perovskite material is mainly filled in the pores of the m-TiO_2_ layer with a thin compact perovskite layer on top. The absorption spectrum and the current density–voltage (*I*–*V*) characteristics along with the performance parameters of the perovskite solar cell device are presented in SI Figure S2. It should be noted that the used two-step sequential interdiffusion technique usually gives slightly lower device performance parameters.

### Transient Absorption (TA) and Transient Electroabsorption (TEA) Measurements

The major technique used here to address the carrier motion is the optical probing of the electric field dynamics performed by TEA measurements. This technique has been previously used for the investigation of the carrier generation and motion dynamics in organic semiconductor layers and organic solar cells.^16,17^ It enables monitoring the motion of photogenerated charge carriers across the investigated layer with an ultrafast, subpicosecond time resolution in the real solar cell architecture.

TA and TEA investigations were performed with a femtosecond transient absorption spectrometer based on the amplified femtosecond laser (Pharos 10-600-PP, Light Conversion Ltd.), operating at a fundamental wavelength of 1030 nm and generating pulses of an ∼230 fs duration at a repetition rate of 50 kHz. The optical measurement scheme is presented in Supporting Information (SI) Figure S1. The excitation wavelength was chosen at 535 nm via a collinear optical parametric amplifier (Orpheus PO15F2L, Light Conversion Ltd.). The measurements were performed at a repetition rate of 4.554 kHz frequency achieved by using the pulse picker. In the case of TA measurements, the optical excitation pulses were chopped at 0.759 kHz frequency by a mechanical chopper synchronized to the electrical output signal of the pulse picker. In the case of TEA measurements, the excitation pulses were delivered continuously, while the electrical pulses were chopped instead. Electrical pulses were generated by means of an arbitrary function generator (Tektronix AFG2021, Tektronix Ltd.), and in this case, the electrical gate mode of the arbitrary function generator, synchronized to the electrical output of the chopper, was used. For the probe beam, spectrally broadened pulses by means of continuum generation in the sapphire crystal were used. The delay time between the excitation and probe light pulses was changed by the optical delay line based on a retroreflector optics mounted on the electromechanical translation stage (Aerotech PRO165LM, Aerotech Ltd.). The detection equipment consisted of a spectrometer (Andor Shamrock SR-500i-B1-R, Andor Technology Ltd.), equipped with a temperature-controlled CCD camera (Andor Newton DU970P-UVB, Andor Technology Ltd.). The reading of the camera was synchronized with the chopper (or, accordingly, with chopped electrical pulses). The TA or TEA spectra were calculated as a difference between the sample absorbance under excitation (or under electrical pulses, respectively) and without.

To avoid sample modifications during our measurements, which are typical for perovskites, the measurements were performed by scanning kinetics very rapidly for several minutes, while to increase the accuracy, the results were averaged over multiple measurement scans.

### Photoluminescence Measurements

The photoluminescence dynamics was measured using a streak camera system (Hamamatsu C5680) with a synchroscan (M5675) module coupled to a spectrometer. A femtosecond Yb:KGW oscillator (Light Conversion Ltd.) produced 80 fs, 1030 nm light pulses at a repetition rate of 76 MHz, which were frequency doubled to 515 nm (HIRO harmonics generator, Light Conversion Ltd.) and used for the sample excitation. The laser pulses were attenuated and focused into an ∼80 μm spot on the sample, resulting in an excitation energy density of approximately 3 nJ·cm^–2^. The maximum time resolution of the streak images was about 3 ps. To avoid the accumulation of photogenerated charge carriers and of the screening of the electric field by mobile ions, the voltage has been applied by 10 ms pulses at a 10 Hz repetition rate. The excitation light was also applied only during the voltage pulse action.

### Multivariate Curve Resolution (MCR) Modeling

The application of the MCR method to the spectroscopic data is based on the assumption that the two-dimensional (wavelength and time) data set can be expressed as a linear combination of *N* spectral components *S*_n_(λ) with their corresponding kinetics *K*_n_(*t*)
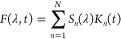
Contrary to the usual global analysis methods, MCR does not assume an exponential time dependence of *K*_n_(*t*); thus, nonexponential dynamics can be uncovered. This is highly beneficial when analyzing disordered inhomogeneous systems, like perovskites. In our analysis, we have used two constraints—nonnegativity and unimodality of kinetics *K*_n_(*t*). The actual shapes for *S*_n_(λ) or *K*_n_(*t*) are uncovered by using the alternating least-squares algorithm to minimize the mean-squared error^18^
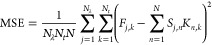


## Results and Discussion

The main experimental tool used in this study was the transient electroabsorption technique, which allows probing the electric field strength in the perovskite layer with subpicosecond time resolution, and based on this information, it enables the characterization of charge carrier drift dynamics. Briefly, the applied voltage together with the built-in electric field creates an internal electric field in the perovskite layer and changes its absorption spectrum due to the electroabsorption (EA) effect. When the sample is excited with ultrashort light pulses, the generated charge carriers drift in the internal electric field, partially screen it, partly discharge the sample capacitance, and weaken the EA spectrum. We will call this weakening as the EA component. From the dynamics of the EA component, we recalculate the weakening of the internal electric field and evaluate the drift dynamics of the photogenerated charge carriers in a similar way as in the case of the integral regime of the conventional TOF technique.^19^

Additionally, the applied reverse voltage accelerates the charge carrier extraction from the photoexcited perovskite into the transport layers and also changes the perovskite absorbance by weakening the conventional transient absorption (TA) due to the photogenerated charge carriers. Since the TA spectrum is dominated by the absorption bleaching (AB) (see Figure 1d), we will call this weakening as the AB component. The dynamics of the AB component represents the voltage-induced carrier extraction. Both EA and AB components contribute to the transient absorbance of MAPI excited by femtosecond light pulses under applied voltage. Fortunately, the two components have significantly different spectral features, as shown in Figure 1d, enabling reliable separation between them. Technically, the investigations were carried out with the femtosecond transient absorption spectrometer described in the Materials and Methods Section. We used it in two different regimes: the conventional transient absorption (TA) regime and the transient electroabsorption (TEA) regime. Figure 1b,c shows the timing of the optical and electrical pulses in both regimes. In the conventional TA regime, the electrical pulses were always applied simultaneously with the probe pulses, while the optical excitation was modulated, and we measured the conventional differential transient absorbance including that under the applied voltage. In the TEA regime, the electrical pulses were modulated, and we measured the difference in the absorbance of the photoexcited sample with and without the applied voltage.

Figure 1d shows the EA and AB spectral components measured for the investigated MAPI solar cell. The EA spectrum was measured in the TEA regime but without optical excitation, while the AB spectrum was measured in the conventional TA regime under a 1 V forward applied voltage compensating the built-in electric field. The TEA investigations were performed using a positive background voltage of 0.6 V, which partially compensates the built-in voltage while the dark current is still weak. It should be noted that the measured TEA spectrum is slightly different from that reported for MAPI, e.g., in ref (20). The short-wavelength side of the TEA spectrum is weakened due to the technical problems related to the low probe beam transmittance in this spectral region. Therefore, we evaluated the intensity of the EA spectrum as a difference between the maximal values of the long wavelength positive and negative EA bands, as shown in Figure 1d. As Figure 1e shows, the intensity of EA increases proportionally to the square of the internal voltage, which is a sum of the applied voltage and the built-in potential, as expected for the quadratic EA effect. Kinetics of the EA intensity was used to recalculate the electric field dynamics induced by the optical excitation. During the TEA measurements, the electric field was applied by short electrical pulses of 20 μs duration because longer electrical pulses weaken the EA spectrum, as demonstrated in Figure 1f. This phenomenon is caused by mobile ions, which partially screen the electric field in the perovskite layer when long electrical pulses are used.

### Carrier Drift and Diffusion

**Figure 2:**
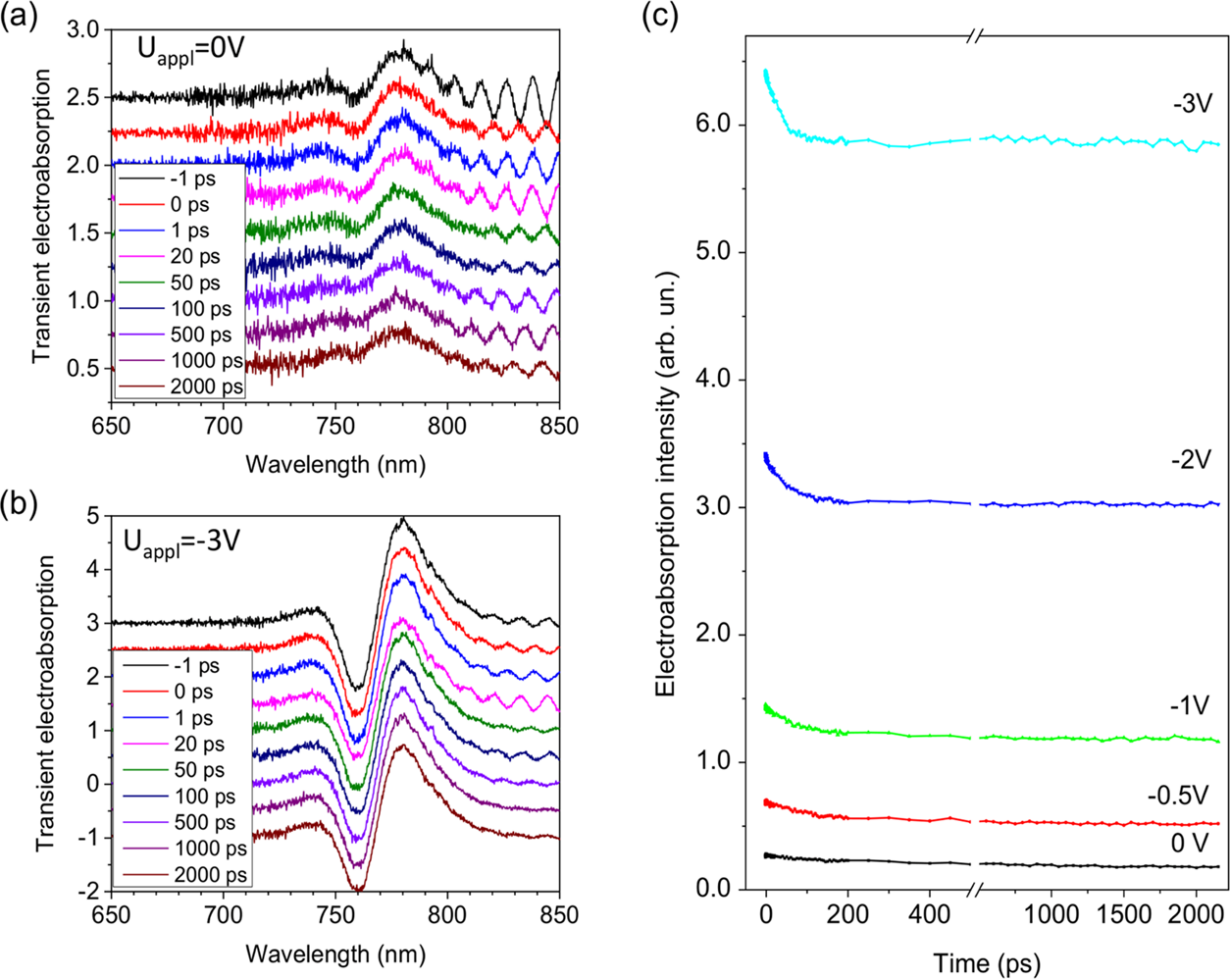
Dynamics of the TEA spectrum measured under 0 V (a) and −3 V (b) electrical pulses under excitation with 0.15 μJ·cm^–2^ intensity, 535 nm pulses. The spectra at different delay times are shifted vertically for clarity; (c) kinetics of the EA intensities at different applied voltages evaluated, as shown in Figure 1d.

We begin the discussion of our experimental results by analyzing the EA dynamics in the photoexcited solar cell. Figures 2a and b show the TEA spectra of the MAPI perovskite solar cell at different delay times after optical excitation by very low intensity (0.15 μJ·cm^–2^) pump pulses, under 0 and −3 V applied voltages. The time-averaged excitation intensity was about 0.6 mW/cm^2^, which is roughly 100 times lower than the intensity of sunlight. TEA spectra under other voltage values are presented in SI Figure S3. The TEA signal at a 0 V voltage appears due to the built-in electric field. At negative delay times, i.e., when the probe pulse precedes the excitation pulse, the TEA spectrum corresponds to the conventional EA spectrum. At positive delays, the TEA spectra slightly weaken with the delay time, indicating weakening of the internal electric field inside the perovskite layer when drifting photogenerated charge carriers partially screen the electric field. The relatively small weakening of the EA spectrum with the delay time indicates that the electric field screening by drifting carriers is insignificant and we can approximately consider that the carriers move in a constant electric field. Under these low excitation intensity conditions, the AB component is weak and, as we will show below, relatively weakly depends on the applied voltage; thus, the EA component strongly dominates in the TEA spectrum. Therefore, TEA dynamics is determined by the EA variations and we can evaluate the EA intensity as a difference between the TEA signal at the maxima of positive and negative bands. Figure 2c shows the EA dynamics at different applied voltages. Taking into account the quadratic dependence of the EA intensity on the internal electric field, we can simply evaluate the dynamics of the internal electric field as being proportional to a square root of the EA intensity.

**Figure 3:**
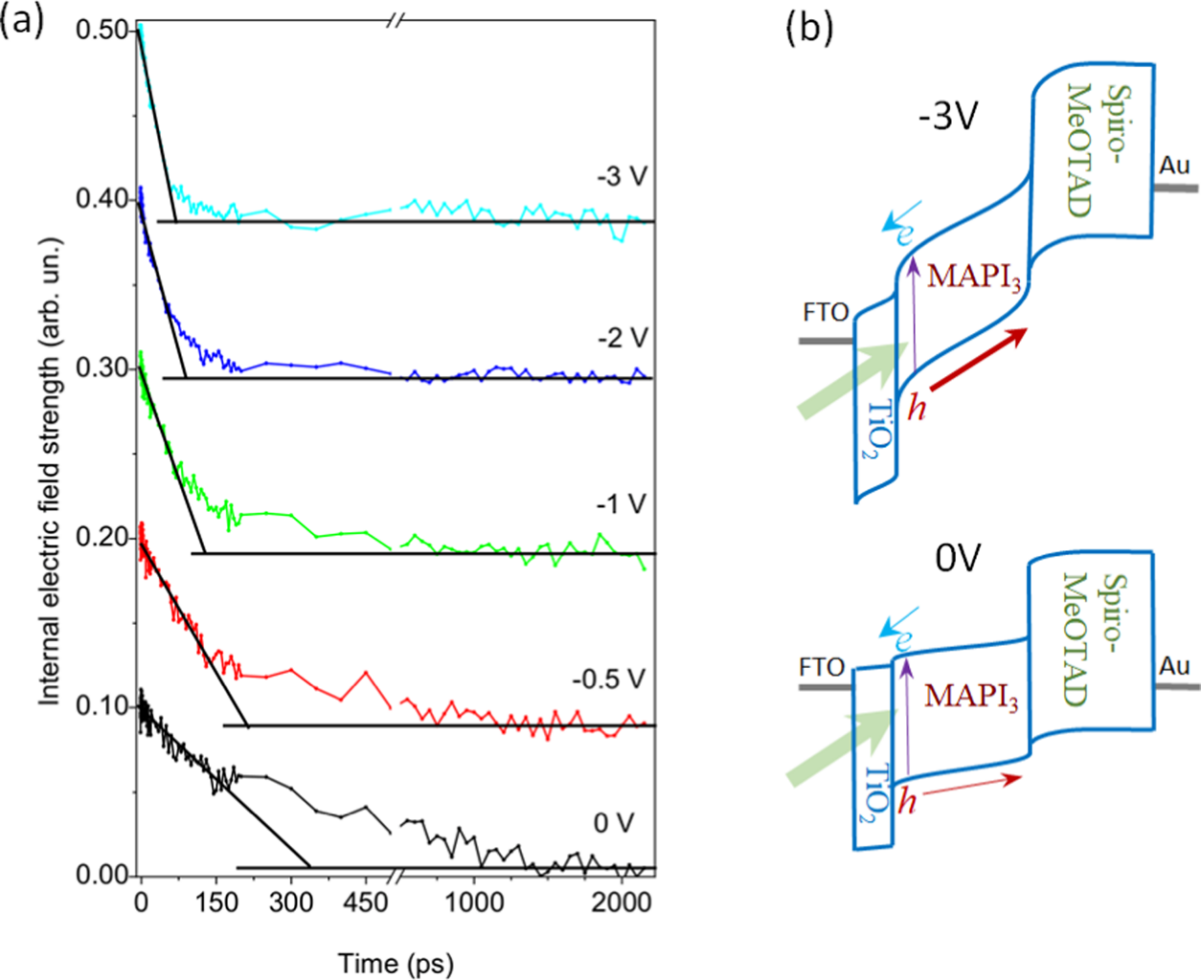
(a) Internal electric field dynamics at different applied voltages after sample excitation by 0.15 μJ·cm^–2^ intensity pulses. For better presentation, the curves are vertically shifted; and (b) band diagram of the MAPI perovskite solar cell at different applied voltages.

Figure 3 shows the evaluated dynamics of the internal electric field after excitation by 0.15 μJ·cm^–2^ excitation pulses obtained at different effective voltages. However, internal electric field values here are presented in arbitrary units because, as will be discussed below, determination of the absolute electric field strength in the m-TiO_2_/perovskite layer is not a trivial task. For better presentation, the curves are shifted vertically. It is noteworthy that the total excitation-induced electric field drop is independent of the applied voltage, as we expect for discharging of the sample capacitance by drifting constant charge (Δ*U* = *Q*/*C*). It indicates that all photogenerated charge carriers indeed drift across the entire thickness of the perovskite layer during an observation time of 2 ns for all values of the applied voltage. The low excitation intensity simplifies the data analysis since the internal electric field changes only by about 5–20% depending on the applied voltage. We can therefore approximately assume that the charge carrier drift takes place in a constant electric field. Depending on the applied voltage, the electric field decreases on a time scale of tens to hundreds of picoseconds while it remains nearly constant for longer times.

The initial decrease is well approximated by a linear function. Our measurement technique closely resembles the integral regime of the time-of-flight (TOF) technique,^19^ yet with more than 3 orders of magnitude higher temporal resolution. Figure 3b shows the supposed band diagrams at different applied voltages. Because of the high optical density of the m-TiO_2_/perovskite layer, the charge carriers are mostly generated close to the positively biased compact TiO_2_ electrode, therefore drifting only a short distance. Therefore, the current is dominated by holes crossing the m-TiO_2_/perovskite and the compact perovskite layers toward the spiro-OMeTAD HTL. The initial linear decay of the electric field indicates that the drifting cloud of holes creates a constant current; thus, the initial drift of the charge carriers may be characterized by a constant mobility. The decrease in the electric field ceases when the photogenerated holes cross the perovskite layer and reach the interface with the HTL. Thus, the intersection of the line approximating the electric field decay with the stabilized electric field value (see Figure 3) gives the duration of charge carrier transit through the perovskite layer τ_tr_, assuming that all charge carriers cross the entire perovskite layer with a constant velocity. This situation approximately occurs at a −3 V applied voltage, while at lower voltages a slower electric field decay phase lasts for hundreds of picoseconds. The linear approximation of the fast decay phase gives τ_tr_ times equal to 300 ps, 210 ps, 135 ps, 90 ps, and 64 ps obtained at 0, −0.5, −1 V, −2, and −3 V applied voltages, respectively.

We can evaluate the carrier mobility as μ = *d*^2^/((*U*_app_ + *U*_built in_)τ_tr_). However, this task is not so trivial because of the difficulty in estimation of the actual internal electric field strength in the perovskite layer. One extreme assumption is that all the internal voltage drops on the m-TiO_2_/perovskite and compact perovskite layers. Assuming also that *U*_built in_ = −1 V leads to a lower limit of the initial hole mobility, which we obtain equal to 3.0, 3.2, 2.7, 2.7, and 2.6 cm^2^·V^–1^·s^–1^ at 0 V, −0.5, −1, −2, and −3 V applied voltages, respectively. The obtained mobility values within evaluation accuracy are remarkably independent of the applied voltage, which indicates that the carrier mobility is independent of the applied voltage and also validates the evaluation procedures.

Another assumption is that the charge carrier densities in our experimental conditions (in the dark, under negative applied voltages) are low in all solar cell layers; therefore, the electric field strengths in different layers are determined by their dielectric constants, as suggested in ref (13)., i.e., inversely proportional to the dielectric constant. Both perovskite and anatase TiO_2_ have very high low-frequency dielectric constants of about 30–60,^21,22^ while a dielectric constant of organic spiro-OMeTAD HTL is about 3. Such an estimation implies that only about 10% of the total internal voltage drops on m-TiO_2_/perovskite and compact perovskite layers. This assumption gives about 10 times higher hole mobility values ranging from about 25 to 31 cm^2^·V^–1^·s^–1^. However, this assumption is also hardly realistic because spiro-OMeTAD is doped and conductive. Under the electric field, the depletion layer may be formed, but its thickness is apparently much smaller than the total layer thickness. Mobile ions may additionally screen the electric field; however, according to Figure 1f, this screening is hardly significant under used short electrical pulses. Therefore, it is more likely that the electric field in perovskite may be reduced at most several times. Consequently, carrier mobility may hardly be larger than about 10 cm^2^·V^–1^·s^–1^, in agreement with previous reports.^23^

Both these limiting values fit within a wide range of carrier mobility values reported for MAPI perovskite, ranging from 0.4 to 71 cm^2^·V^–1^·s^–1^ according to ref (4). A similar mobility value of 39 cm^2^·V^–1^·s^–1^ was also evaluated from the carrier diffusion analysis in the Cs_0.07_Rb_0.03_FA_0.765_MA_0.135_PbI_2.55_Br_0.45_ perovskite solar cell.^24^ Considering the obtained mobility values, two important aspects should be taken into account. First, the m-TiO_2_/perovskite layer consists of about two parts of TiO_2_ and one part of perovskite by volume, as estimated by Zhang et al.^25^ Thus, the carrier mobility in such a mesoscopic structure is expected to be lower than in pure perovskite. Second, most publications report mobility values obtained by steady-state or low time-resolution techniques. On the other hand, our technique has high time resolution, and the abovementioned hole mobility values correspond to the initial mobility measured during the initial 100–200 ps after carrier photogeneration. At longer times, the mobility may decrease due to the carrier trapping or the presence of some structural barriers, such as perovskite grain boundaries;^26^ thus, the initial mobility may be higher than its steady-state value. Such a decrease was found to be very significant, by several orders of magnitude, in organic semiconductors.^27^ The slow carrier drift phase may be considered a signature of the decreasing hole mobility, which may be caused by the trapping/detrapping of a fraction of holes, or by the presence of energy barriers that hinder carrier motion.^12,26^ At strong electric fields, these processes, however, are less important because the carriers may cross the perovskite layer faster than they are trapped, or traps and barriers may be easily surmounted by the assistance of a strong electric field. At lower electric fields, the presence of the large slowly decreasing electric field component (curves at 0 and −0.5 V in Figure 3) shows that a large fraction of holes are trapped during the first 100–200 ps after photoexcitation. However, the traps are apparently shallow because almost all photogenerated holes cross the perovskite layer within about 1 ns. The traps thus just reduce the carrier mobility.

The carrier motion is driven not only by their drift but also by diffusion. The question regarding the role of both processes is still under debate.^13^ We can evaluate the diffusion coefficient by the Einstein–Smoluchowski equation *D* = μ*k*_B_*T*/*q*, where μ is the carrier mobility, *k*_B_ is Boltzmann’s constant, *T* is the absolute temperature, and *q* is the elementary charge. For the two evaluated limiting mobility values of 3.5 and 10 cm^2^·V^–1^·s^–1^, we obtain diffusion coefficient values of about 0.09 and 2.5 cm^2^·s^–1^, respectively. We can estimate the hole diffusion time through the *d* = 300 nm thickness TiO_2_/perovskite layer as τ_dif_ = *d*^2^/*D*. We again obtain two extreme values of about 10 and 2.5 ns. This very rough evaluation shows that carrier drift and diffusion probably play comparable roles under short circuit conditions. However, in the case of steady-state cell operation under real conditions at a voltage of maximum power point, the internal electric field strength should be significantly weaker or even almost completely screened by ions and photogenerated charge carriers prolonging the charge carrier drift time to several tens of nanoseconds. Thus, this estimation leads to the conclusion that carrier diffusion apparently prevails over drift in real operating solar cell conditions, in agreement with previous publications.^13,28^

### Carrier Extraction

**Figure 4:**
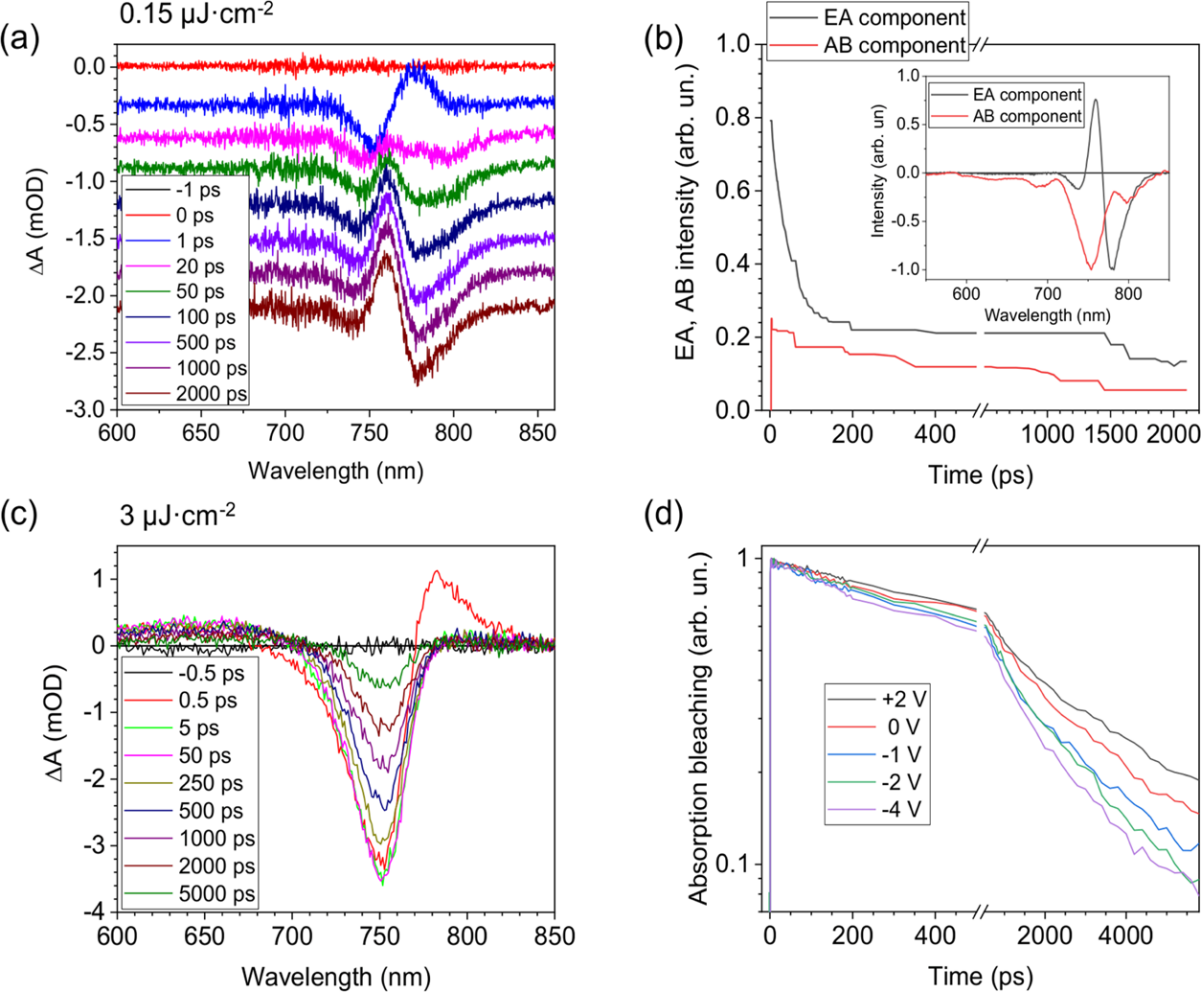
(a) Conventional TA spectra measured at a −3 V applied voltage and a 0.15 μJ·cm^–2^ excitation intensity; the spectra are vertically shifted; (b) kinetics of the EA and AB components obtained by the multivariate curve resolution of data presented in plot (a); (c) TA spectra at a −2 V voltage and a 3 μJ·cm^–2^ excitation intensity; and (d) kinetics of the spectrally integrated absorption bleaching measured under a 3 μJ·cm^–2^ excitation intensity and different applied voltages.

Another important process in perovskite solar cells is the carrier extraction from the perovskite layer. Conventional transient absorption is a convenient tool to probe the carrier extraction dynamics. We can reasonably assume that the intensity of the absorption bleaching is approximately proportional to the carrier density inside the perovskite film, at least at low excitation intensities. Thus, to evaluate the carrier kinetics, we performed additional measurements in the conventional transient absorption regime using the same experimental conditions as for the TEA measurements. The time-dependent TA spectra at a −3 V applied voltage and a 0.15 μJ·cm^–2^ excitation intensity are presented in Figure 4a. These spectra also reveal an interplay between the time-dependent AB and EA components under the applied voltage. In this measurement regime, the two TA components give comparable contributions; therefore, their dynamics cannot be evaluated from the data in a simple way. To this end, we have performed computational decomposition of the experimental data into the two components. Standard global analysis algorithms assume exponential processes, while our investigated processes are far from exponential. Therefore, we applied our original implementation of the multivariate curve resolution algorithm,^29,30^ which we have already used for modeling processes in organic solar cells.^31^ This algorithm produces a certain number of spectral components with (i.e., nonexponential) time evolutions (see the Experimental section for a more detailed explanation). Figure S4 in the SI demonstrates the ability of the model to reproduce the TA dynamics with AB and EA spectral components. Figure 4b shows the kinetics of the obtained two components, and the inset in Figure 4b shows their spectral shapes. The decay of the EA component is very similar to the decay of the EA signal obtained in TEA mode measurements shown in Figure 2c, while the AB component shows a much slower decay. It indicates that the carrier extraction from the perovskite layer, determining the decay of the absorption bleaching, is much slower than the carrier drift determining EA dynamics.

Nevertheless, the accuracy of such decomposition of the spectral components was too low under lower applied voltages at the weak optical excitation intensity, as used in the TEA investigations. It was insufficient to perform a detailed analysis of the carrier decay and their voltage-induced extraction. Therefore, we used a 20 times higher excitation intensity (3 μJ·cm^–2^) when absorption bleaching is much stronger and dominates over the EA signal. Figure 4c shows the evolution of the TA spectrum measured under a −3 V applied voltage. TA spectra at other used applied voltage values are presented in SI Figure S5. Indeed, visually this spectrum closely resembles the spectral shape of the AB component. In this case, we can evaluate the absorption bleaching dynamics under different applied voltages relatively easily from the integrated transient absorption spectra, reasonably assuming that the applied voltage causes a shift of the absorption bands but does not change their intensities. The obtained absorption bleaching kinetics is very similar to that obtained at a very low excitation intensity of 0.15 μJ·cm^–2^ (see SI Figure S6 for comparison). It shows that the increased excitation intensity still was low enough to avoid significant nonlinear processes that could change the carrier decay kinetics.

As Figure 4d shows, the decay of absorption bleaching becomes faster under applied negative voltages, which is due to a faster charge carrier extraction. Importantly, the carrier decay is much slower compared to the carrier drift kinetics discussed above. In fact, the carrier concentration decreases only by about 10% during the first 100 ps, while photogenerated holes almost completely drift through the TiO_2_/perovskite layer during this time at negative applied voltages. This shows that the extraction of holes into the spiro-OMeTAD HTL as well as the extraction of electrons into TiO_2_ is much slower than the drift of the holes, which, in turn, suggests that the holes under the applied negative voltage localize next to the HTL layer during hundreds of picoseconds, while their extraction into the HTL lasts more than 1 nanosecond. It is difficult to exactly evaluate the hole extraction time because transient absorption kinetics accounts also for electron extraction, bimolecular recombination, and extraction of trapped carriers. Nevertheless, a significantly slower transient absorption decay in comparison with the evaluated carrier drift times suggests that the hole extraction is not only limited by their drift and diffusion, but also that hole transfer over the perovskite/spiro-OMeTAD interface is an additional constraint. A similar conclusion has also been derived for the hole extraction through the MAPI_3_/PEDOT-PSS interface.^32^ Large differences in hole extraction times have been reported in the literature, ranging from subpicoseconds in early ultrafast spectroscopic studies^6,33^ to several nanoseconds in more recent evaluations.^24,34^ Electron extraction times also vary within a similar range.^35^ Typically, conclusions on the carrier extraction rates were made from the photoluminescence quenching results when the PL decay rate is determined by both carrier diffusion and interface transfer rates. Moreover, as will be demonstrated below, the PL decay time may be additionally shortened by the spatial separation of the electron and hole distributions. Our technique enabled us to disentangle the hole transport and extraction processes. Consequently, our result shows that carrier transfer through the perovskite/HTL interface is an important limiting factor for carrier extraction and should be considered by analyzing the performance of perovskite solar cells. A limited carrier transfer rate reduces the total carrier extraction rate and, thus, increases the carrier density and recombination losses in the operating solar cell. The carrier transfer rate is, however, not important for the open circuit voltage when carrier extraction does not take place. It can also hardly significantly influence the short circuit current since then carrier density and recombination losses are insignificant. However, its influence on the fill factor and current at the maximal power point may be quite significant. As was discussed, the carrier transport then mainly occurs by their diffusion taking place during several or about tens of nanoseconds. Additional carrier transfer to the transport layer time of the order of 1 ns may increase the total carrier residence within the perovskite layer time by several or even tens of percent, increasing the carrier density and additional recombination losses by the same order of magnitude. On the other hand, the extraction rate may significantly depend on the properties of the perovskite film surface and consequently on the film preparation protocols and conditions.

### Photoluminescence Properties

**Figure 5:**
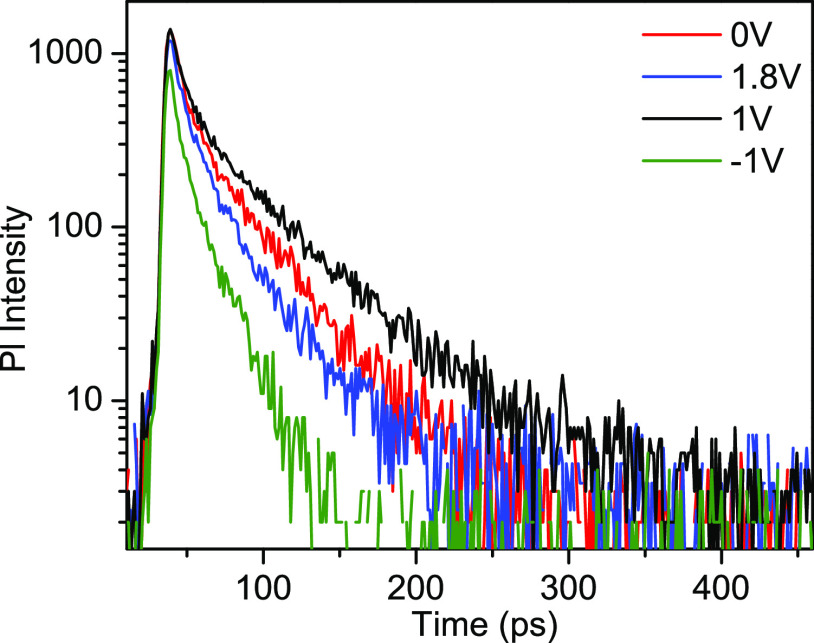
Photoluminescence decay kinetics at different applied voltages under sample excitation by about 100 nJ·cm^–2^, 515 nm subpicosecond pulses at a 76 MHz repetition rate.

Now, we will demonstrate that although conventional time-resolved photoluminescence measurements of perovskites do not provide direct information on the carrier extraction dynamics, they do provide useful insight into the spatial distribution of carriers. Typically, the photoluminescence of MAPI films decays on a time scale of nanoseconds or even microseconds, depending on their fabrication procedures.^36,37^ The PL decay is usually much faster in solar cells, which has been attributed to carrier extraction. Indeed, the TA kinetics shows that the carrier concentration in a solar cell decays on several nanosecond time scales (see Figure 4b,d), i.e., much faster than typically seen in perovskite layers. For better comparison of the TA and PL decay kinetics, we have measured the PL kinetics with a high (about 3 ps) time resolution. Figure 5 shows the spectrally integrated PL kinetics of the investigated solar cell. The measured PL decay kinetics appears to be much faster than typically observed for MAPI_3_ perovskites and even solar cells. This is partly due to the high time resolution and narrow time window (∼2 ns) of our measurements. We were able to observe and resolve the initial fast PL decay component, while the slow component, which lasts for hundreds of nanoseconds, is not observed in our measurements due to the narrow time window and high excitation pulse repetition rate. The PL decays even much more rapidly than the TA. During the initial 100 ps, the PL decays by 1–2 orders of magnitude depending on the applied voltage, while absorption bleaching decreases only by about 10%. The slowest PL decay is observed at +1 V, which is close to the open circuit voltage, when the electric field in the perovskite layer is absent or weak. The PL decay rate becomes much faster under the negative applied voltage when we expect faster carrier extraction, which is in agreement with the TA kinetics presented in Figure 4d. Moreover, the PL decay depends more significantly on the applied voltage than TA. The positive applied voltage (+1.8 V) also accelerates the PL decay but it slows down the TA decay because it prevents carrier extraction. All of these differences imply that TA and PL decays are governed by different processes.

The PL relaxation dynamics and its dependence on the applied voltage are in good agreement with the carrier drift picture described above. Due to the strong perovskite absorbance at a 535 nm excitation wavelength, electrons and holes are photogenerated close to the compact TiO_2_ layer. Under the applied voltage, charge carriers move in opposite directions and their “clouds” spatially separate. The spatial separation reduces their recombination rate and correspondingly the PL intensity. Therefore, the PL decays even faster than the carrier drift across the TiO_2_/perovskite layer. One may argue that the PL and TA should decay simultaneously at a +1 V applied voltage when it compensates for the built-in potential. Nevertheless, the applied voltage can hardly completely compensate for the internal electric field. Spatial charges created by mobile ions, Schottky-type junctions, trapped charge carriers, etc., create local electric fields^13^ that may be sufficient to separate charge carriers. Different electron and hole diffusion rates may also cause their spatial separation even in the absence of electric fields. One may also expect that the spatial separation of electrons and holes and, thus, the PL decay kinetics should also be very sensitive to the excitation conditions. For example, sample excitation by longer wavelength light, which is weakly absorbed by perovskite, may result in more homogeneous carrier generation throughout the entire perovskite layer and, thus, less significant carrier separation effects.

## Conclusions

We addressed charge carrier motion dynamics in an archetypical MAPI perovskite solar cell (PSC) by a combination of conventional ultrafast transient absorption (TA), transient electroabsorption (TEA), and time-resolved photoluminescence (PL) techniques. The TEA reveals electric field screening by the photogenerated drifting holes. The hole drift across the ∼300 nm thick m-TiO_2_/perovskite layer under short circuit conditions was found to take place during hundreds of picoseconds and dominates over diffusion, while at applied voltages closer to the open circuit conditions, carrier diffusion apparently dominates. However, carrier extraction revealed by TEA and TA techniques is slower. It takes place during more than 1 ns and weakly depends on the applied voltage, which indicates that carrier extraction at the reverse voltage and partly at short circuit conditions is more limited by the properties of the interface between perovskite and carrier transport layers than by carrier drift and diffusion. PL decay in PCS under our experimental conditions is very fast, during tens of picoseconds; thus, it is much faster than the decay of carrier concentration. Its dynamics thus reveals the spatial separation of electron and hole clouds. A combination of the three experimental techniques enabled clarification and characterization of the main electronic processes in perovskite solar cells. We believe that this better understanding will contribute to the further development of PSCs.

## Supporting Information Available

Experimental scheme of transient electroabsorption measurements; absorption spectrum and *J–V* characteristics of the solar cell; time evolutions of the transient electroabsorption and conventional transient absorption spectra measured at different voltages; and modeled transient absorption kinetics (PDF)
