# Supplementary material for: Direct Tracking of Charge Carrier Drift and Extraction from Perovskite Solar Cells by Means of Transient Electroabsorption Spectroscopy
Source: ACS Appl Electron Mater. 2023 Jan 11;5(1):317–26. doi: 10.1021/acsaelm.2c01346 (PMC11008527; doi:10.1021/acsaelm.2c01346)
Supplement: Supplementary file 1 — el2c01346_si_001.pdf [file el2c01346_si_001.pdf]

Supporting Information

Direct tracking of charge carrier drift and extraction from perovskite solar cells by means of transient electroabsorption spectroscopy

Vidmantas Jašinskas[1]*, Marius Franckevičius*[1,*]*, Andrius Gelžinis*[1,2]*, Jevgenij Chmeliov*[1,2] *and Vidmantas Gulbinas*[1,2,*]

[1] Department of Molecular Compound Physics, Center for Physical Sciences and Technology, Saulėtekio av. 3, Vilnius, LT-10257, Lithuania

[2]Institute of Chemical Physics, Faculty of Physics, Vilnius University, Saulėtekio av. 9, Vilnius, LT-10222, Lithuania

[*]e-mail: marius.franckevicius@ftmc.lt; vidmantas.gulbinas@ftmc.lt

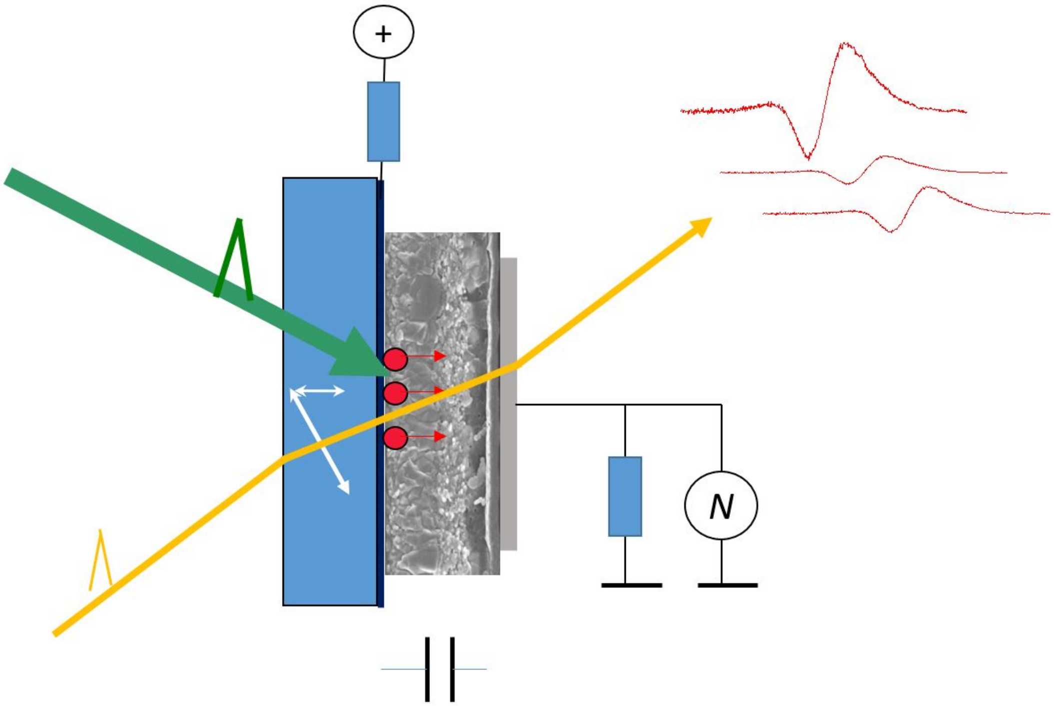

Figure S1. Optical and electrical scheme of transient electroabsorption measurements. Active layers of perovskite solar cell sandwiched between FTO and gold electrodes form electrical capacitor, which is charged prior to optical excitation. Excitation laser pulse (green) photogenerates charge carriers close to FTO electrode. Photogenerated holes move through the sample and partly discharge the capacitor. The electric field inside m-TiO_2 /perovskite layer is probed by the p-polarized white light continuum pulse, which propagates through the sample at about 45 deg. angle. Parallel to the applied electric field electric component of the probe light filed probes the electroabsorption spectrum of perovskite, which gradually decreases with the probe pulse delay when moving holes reduce the internal electric field.

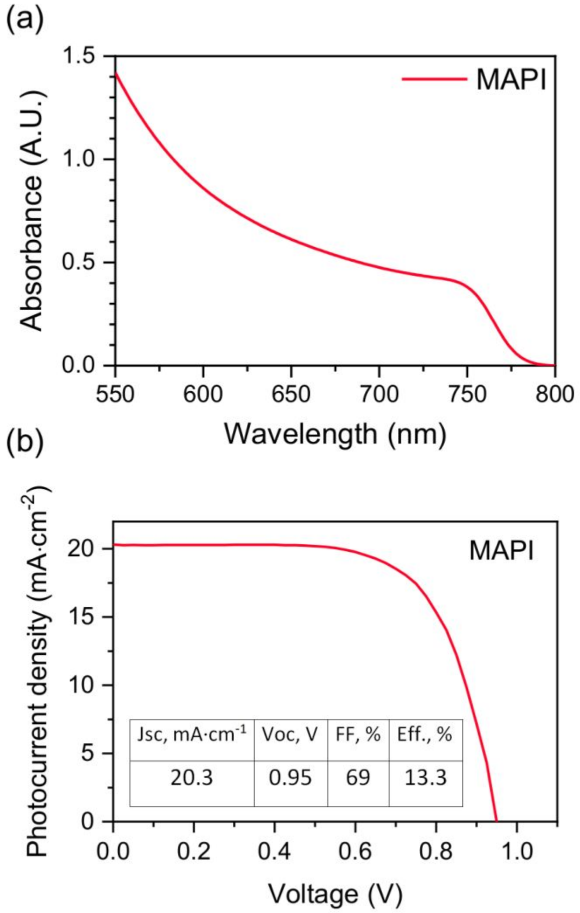

Figure S2. Absorption spectrum (a) and I-V characteristics (b) of the investigated MAPI solar cell.

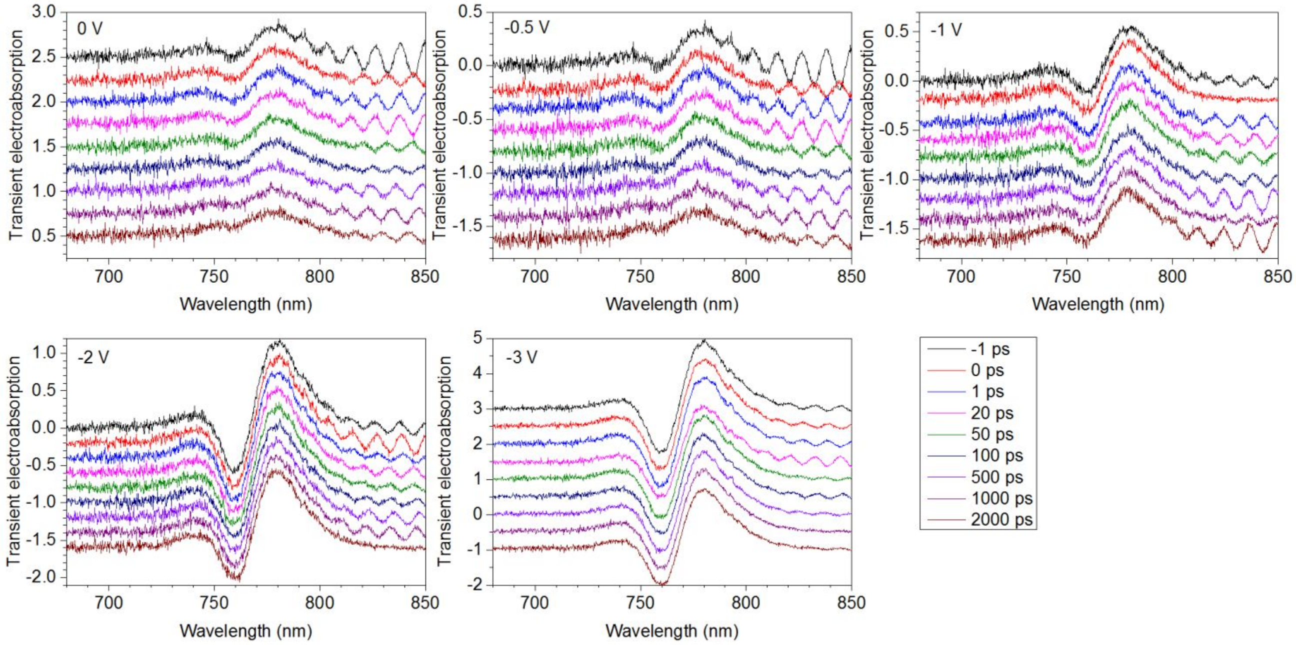

Figure S3. Time evolutions of the TEA spectra of MAPI perovskite solar cell at different applied voltages under 0.15 μJ·cm^{-2} excitation intensity.

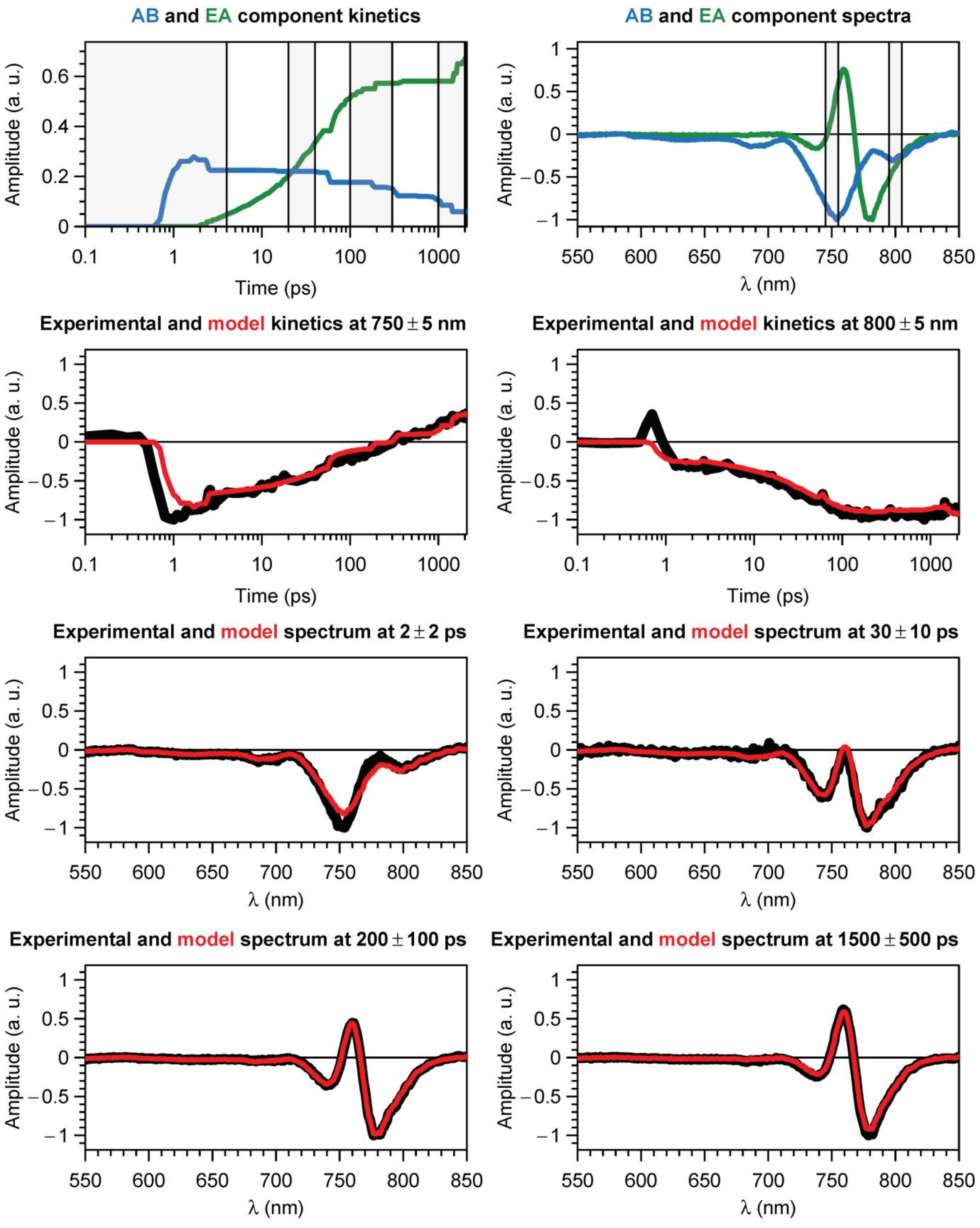

Figure S4. Modelled TA kinetics measured at –3 V applied voltage and 0.15 μJ·cm^{-2} excitation intensity. Top left plot shows the obtained kinetics of the AB and EA components. Top right plot shows spectra of the AB and EA components. Kinetics and spectra presented below show agreement between modelled (red) and experimental (black) data.

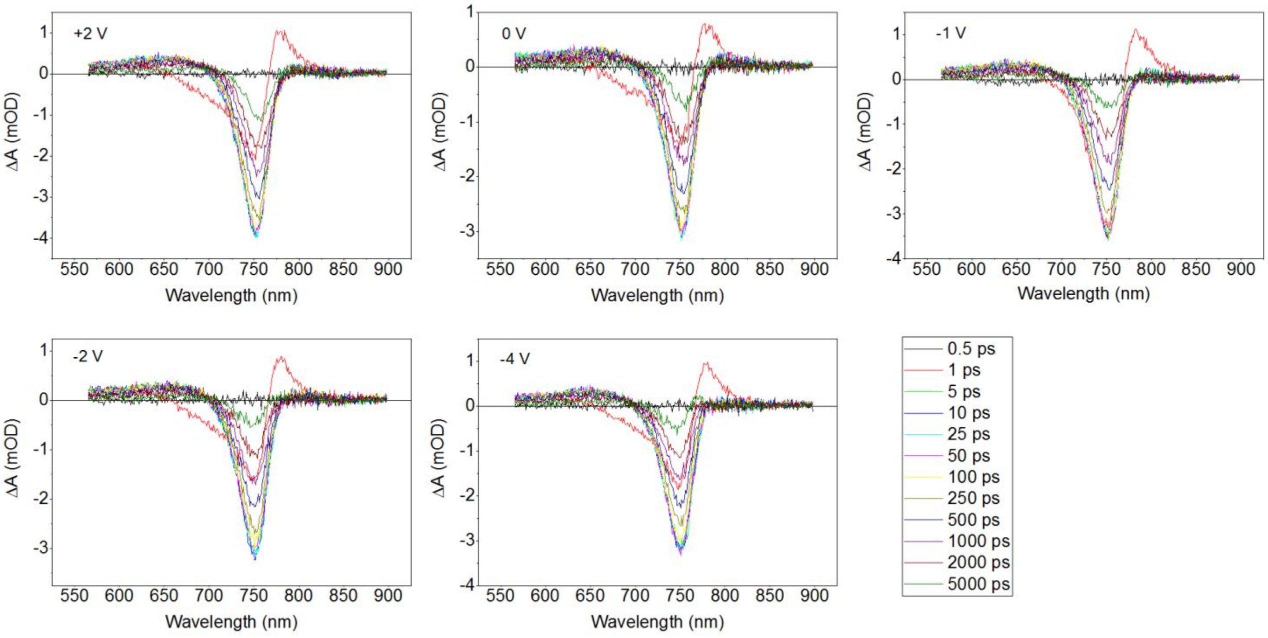

Figure S5. Conventional TA spectra dynamics measured at different applied voltages under 3 μJ·cm^{-2} excitation intensity.

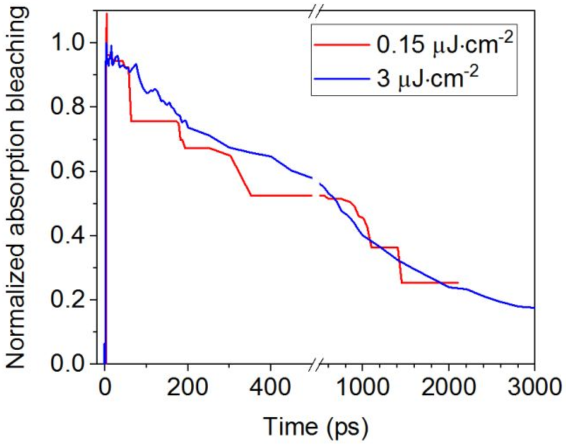

Figure S6. Comparison between the absorption bleaching kinetics at –3V applied voltage obtained by the multivariate curve resolution algorithm applied for the TA data at 0.15 μJ·cm^{-2} (red curve) and absorption bleaching at 3 μJ·cm^{-2} obtained by integration of TA spectra (blue curve).
